# Supplementary figures and images for: Notch signaling is a critical initiator of roof plate formation as revealed by the use of RNA profiling of the dorsal neural tube
Source: BMC Biol. 2021 Apr 23;19:84. doi: 10.1186/s12915-021-01014-3 (PMC8063321; doi:10.1186/s12915-021-01014-3)

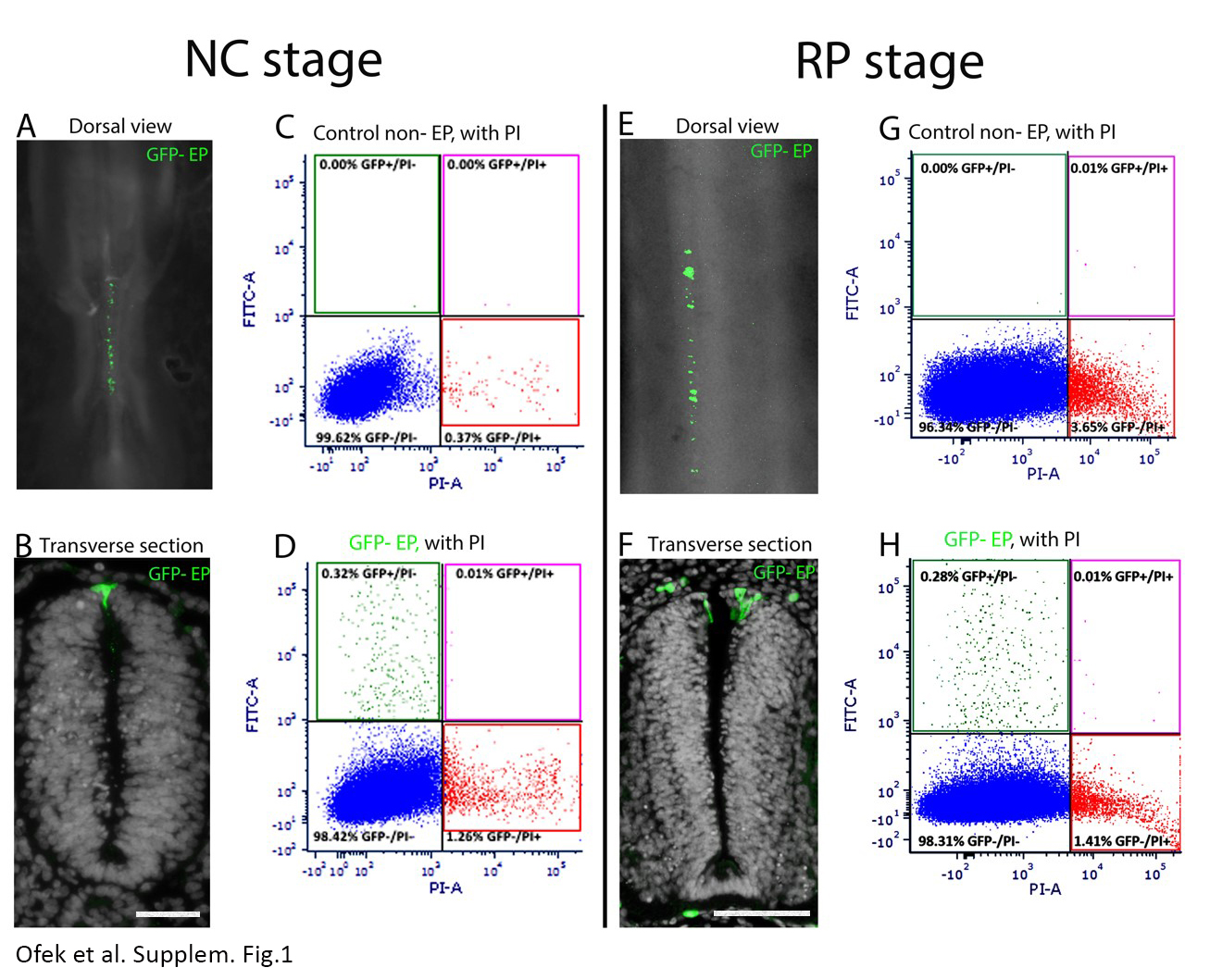

Supplement: Supplementary file 1 — Additional file 1: Fig. S1. Labeling of the dorsal NT at NC and RP stages followed by FACS analysis. Focal electroporations of GFP-DNA were directed to the dorsal NT at NC and RP stages. Embryos were sacrificed 6-8hr later to isolate premigratory NC progenitors prior to the onset of EMT. (A,E) dorsal views of live embryos showing restricted labeling of the dorsal midline domain at both stages. (B,F) Focal dorsal labelings confirmed in transverse sections. Green cells outside the NT in F represent autofluorescence of blood cells. (C,D,G,H) FACS purification of GFP-labeled cells. Note absence of GFP+ cells in control non-electroporated samples (C and G) and very low proportions of propidium iodide (PI)+ dead cells in both control and electroporated cases. PI+ cells were excluded by gating and GFP+/PI- cells were collected (D,H). As expected from focal transfections, the percentage of live, labeled cells (GFP+/PI-) was 0.32% and 0.28% of the total input for NC and RP, respectively. Abbreviations, EP, electroporation, FITC, fluorescein isothiocyanide. Bar= 50μm. [file 12915_2021_1014_MOESM1_ESM.jpg]

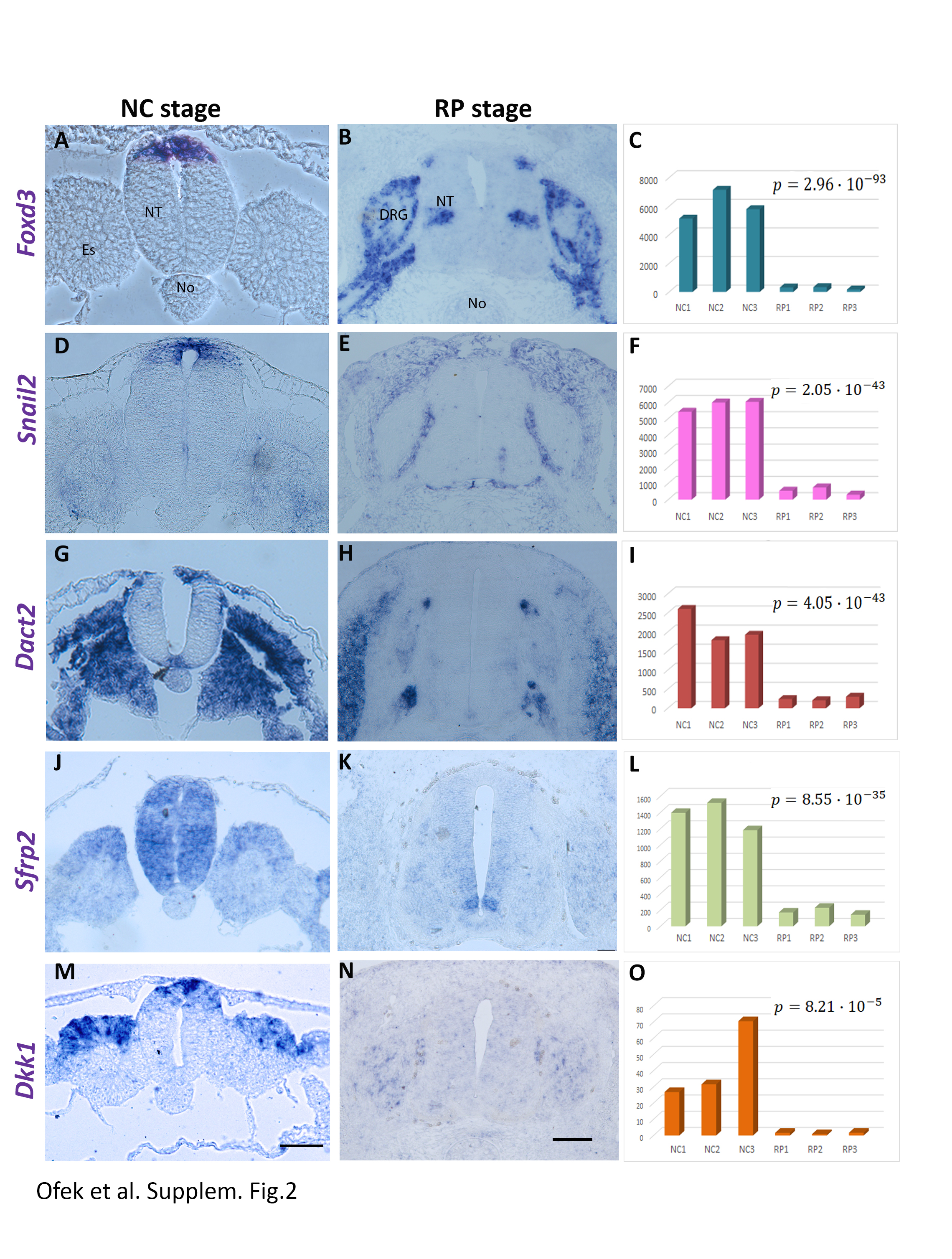

Supplement: Supplementary file 4 — Additional file 4: Fig. S2. In situ hybridization (ISH) for selected genes downregulated in RP compared to NC. (A, D, G, J, M) Transcripts expressed in the dorsal tube at the NC but not RP stage (B, E, H, K, N). (C, F, I, L, O) Quantification of gene expression levels stemming from the transcriptome analysis. NC1–3 and RP1–3 represent experimental triplicates. Note positive correlation between ISH and transcriptome results. In each chart, the B-H adjusted p value is indicated. Abbreviations, DRG, dorsal root ganglion, Es, epithelial somite, NT, neural tube, No, notochord. Bar = 50 μm. [file 12915_2021_1014_MOESM4_ESM.jpg]

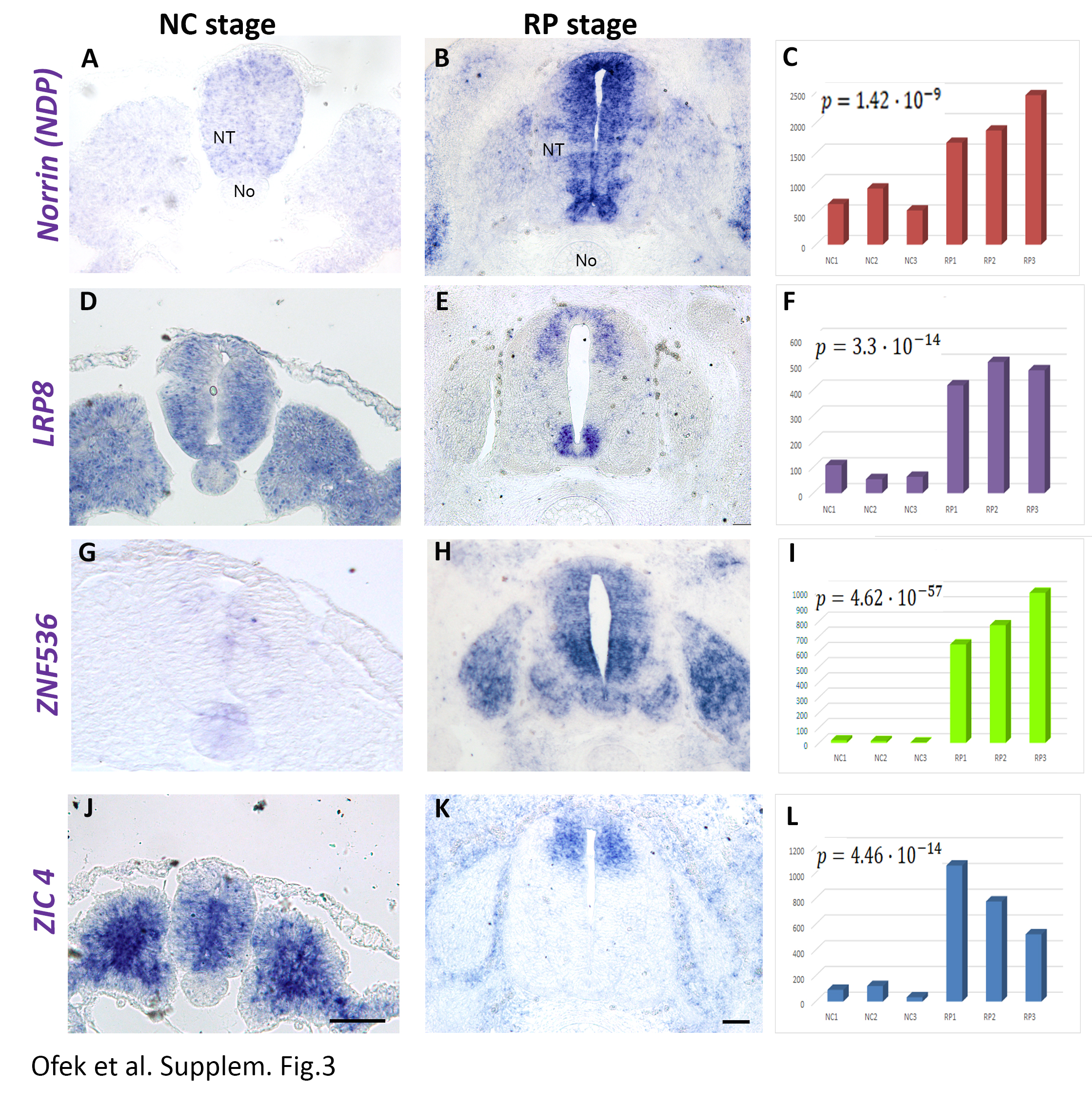

Supplement: Supplementary file 5 — Additional file 5: Fig. S3. ISH for selected genes expressed in the RP as well as in more ventral domains of the NT. (A, D, G, J) ISH at NC and RP (B, E, H, K) stages, respectively. (C,F,I,L) Quantification of gene expression levels stemming from the transcriptome analysis. In each chart, the B-H adjusted p value is indicated. Note at RP stage the extended expression of transcripts beyond the RP domain. Abbreviations, NT, neural tube, No, notochord. Bar= 50μm. [file 12915_2021_1014_MOESM5_ESM.jpg]

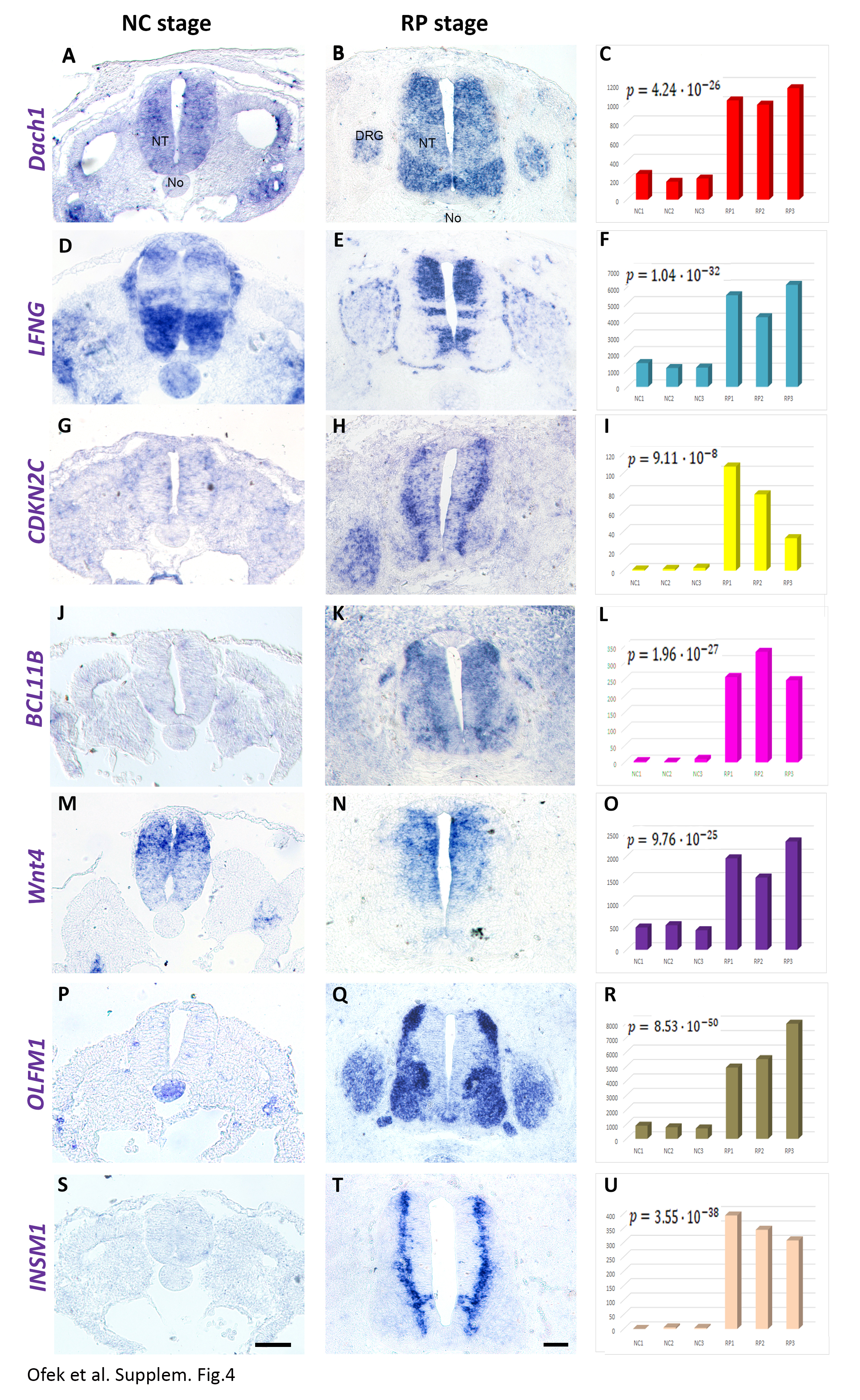

Supplement: Supplementary file 6 — Additional file 6: Fig. S4. ISH for selected “non-RP” genes upregulated at the RP stage but not transcribed in RP. (A,D,G,J,M,P,S) ISH at the NC stage. (B,E,H,K,N,Q,T) ISH at the RP stage. (C,F,I,L,O,R,U) Quantification of gene expression levels. In each chart, the B-H adjusted p value is indicated. Note in the middle column, the expression of transcripts throughout the NT except for the RP in spite of transcript levels being upregulated in RP compared to NC (right column). Bar = 50 μm. [file 12915_2021_1014_MOESM6_ESM.jpg]

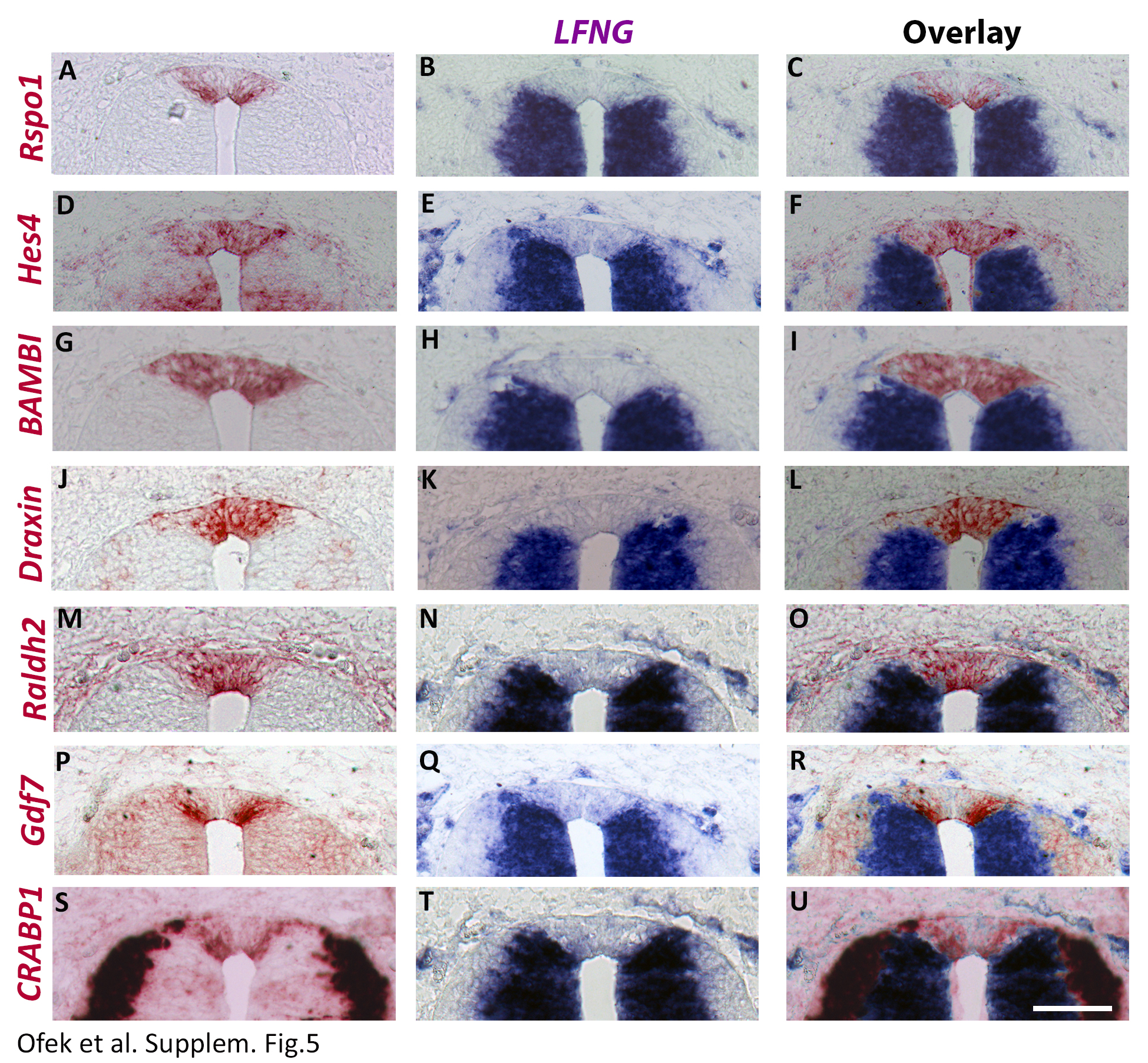

Supplement: Supplementary file 7 — Additional file 7: Fig. S5. ISH of adjacent sections with selected RP markers in combination with the “non-RP” marker lunatic fringe (LFNG). (A,D,G,J,M,P,S) ISH for RP markers. (B,E,H,K,N,Q,T) ISH for LNFG. (C,F,I,L,O,R,U) Overlay of the precedent showing complementary marker expression. Bar= 50μm. [file 12915_2021_1014_MOESM7_ESM.jpg]

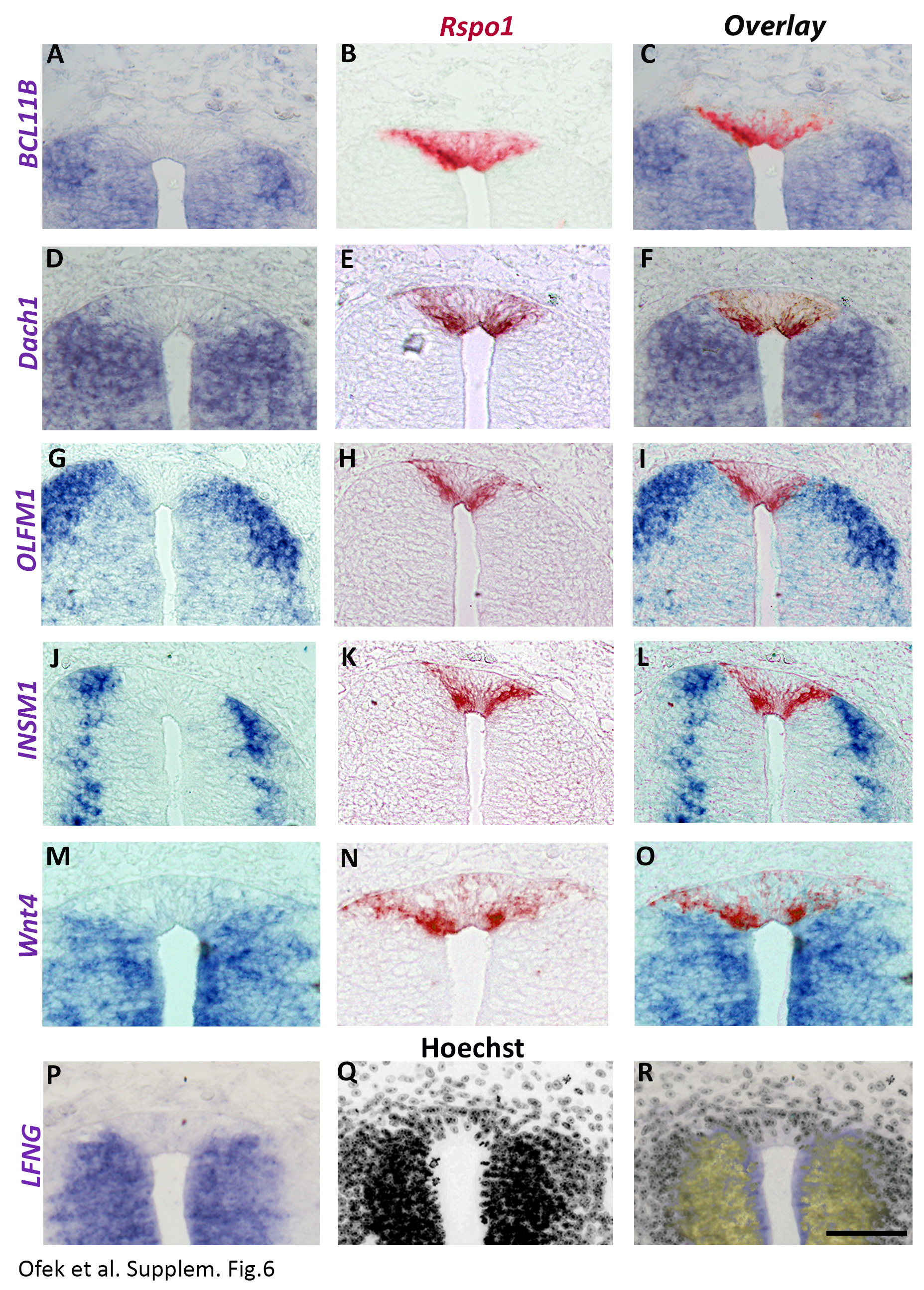

Supplement: Supplementary file 8 — Additional file 8: Fig. S6. ISH of adjacent sections with selected “non-RP” genes in combination with Rspo-1. (A,D,G,J,M) ISH for non-RP markers. (B,E,H,K,N) ISH for Rspo1. (C,F,I,L,O) Overlay of the precedent showing complementary marker expression. (P,Q,R) Combined ISH for LFNG and Hoechst nuclear staining. Bar = 50 μm. [file 12915_2021_1014_MOESM8_ESM.jpg]

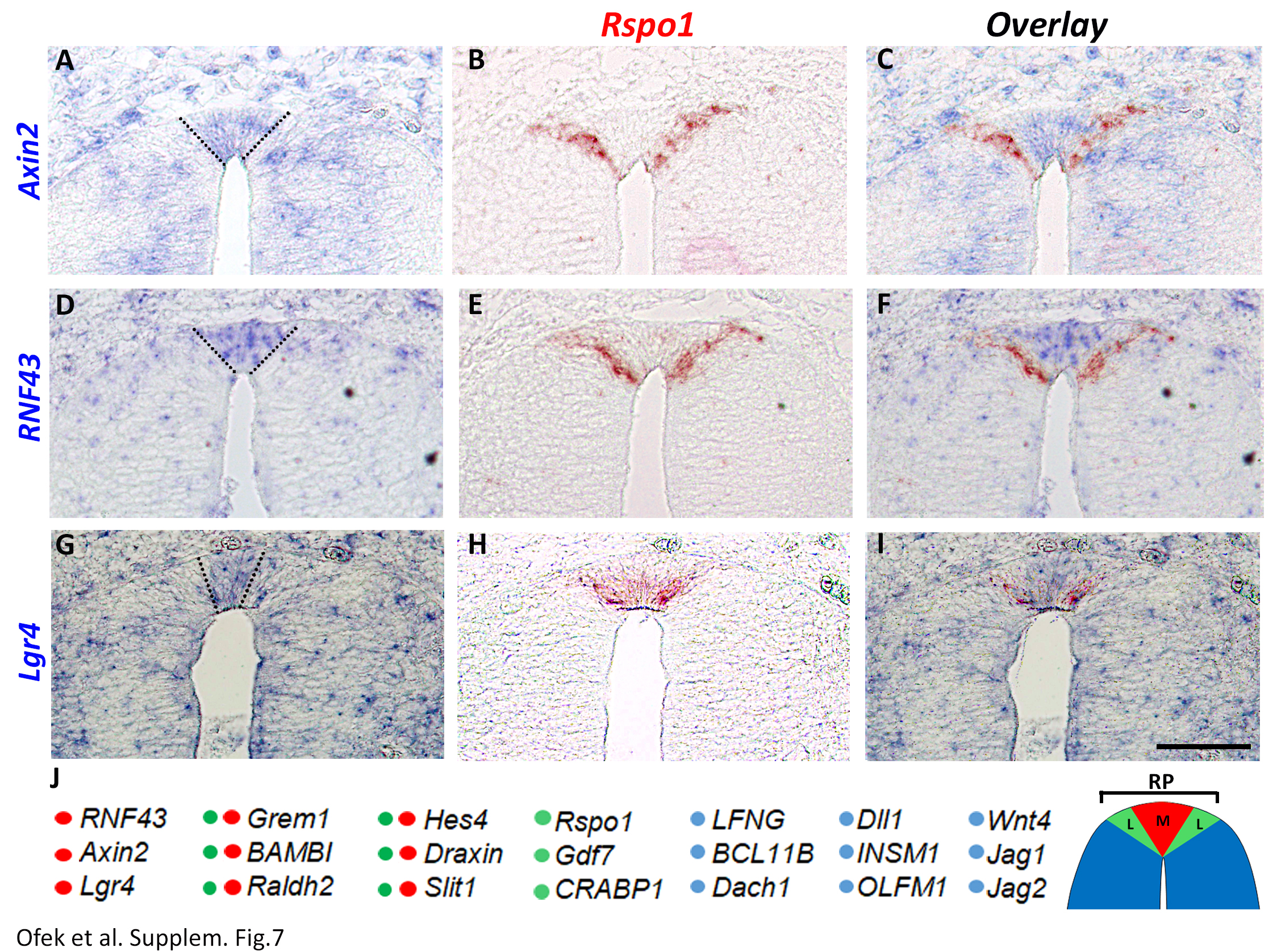

Supplement: Supplementary file 9 — Additional file 9: Fig. S7. Molecular heterogeneity within the RP. (A,D,G) Expression of three genes in the central domain of the RP (delimited by dotted lines). (B,E,H) Rspo1 is preferentially expressed in the RP periphery. (C,F,I) Combination of adjacent sections showing complementary expression of the above. (J) Schematic representation of the expression pattern of several genes upregulated at the RP stage to either its medial (M, red), lateral (L, green), to both domains (green+red) or to NT regions except for the RP (blue). Bar= 50μm. [file 12915_2021_1014_MOESM9_ESM.jpg]

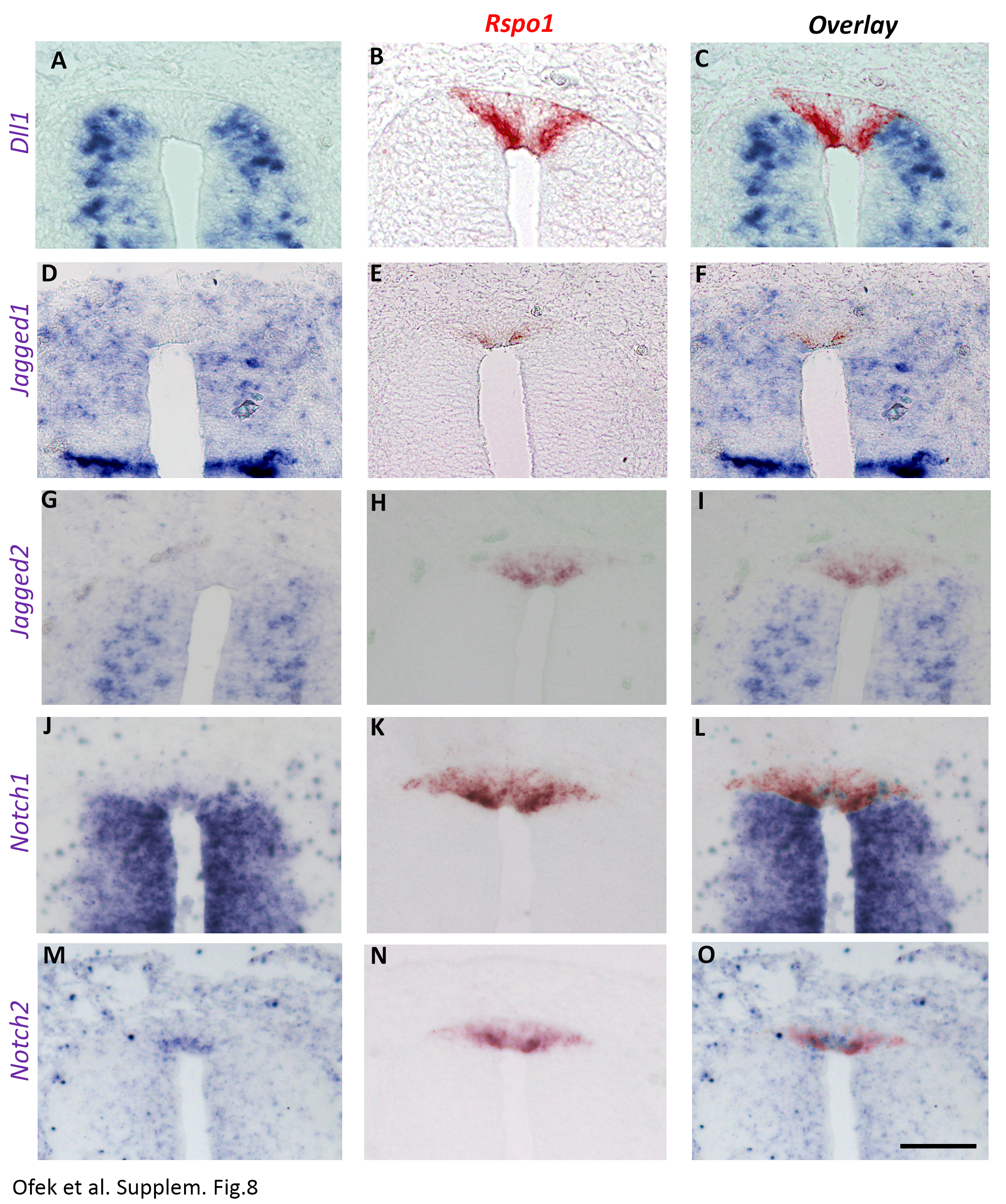

Supplement: Supplementary file 10 — Additional file 10: Fig. S8. Expression patterns of Notch ligands and receptors. (A,D,G,J,M) Expression of three Notch ligands (A,D,G) and the Notch1 and 2 receptors (J,M) at the RP stage. (B,E,H,K,N) Rspo1 expression in adjacent sections. (C,F,I,L,O) Overlay of the above. Note that the dorsal limit of Dll1, Jagged1 and Jagged2 mRNA expression corresponds to the ventral limit of the RP. Notch1 and Notch2 mRNAs are expressed apically in the RP domain. Bar= 50μm. [file 12915_2021_1014_MOESM10_ESM.jpg]

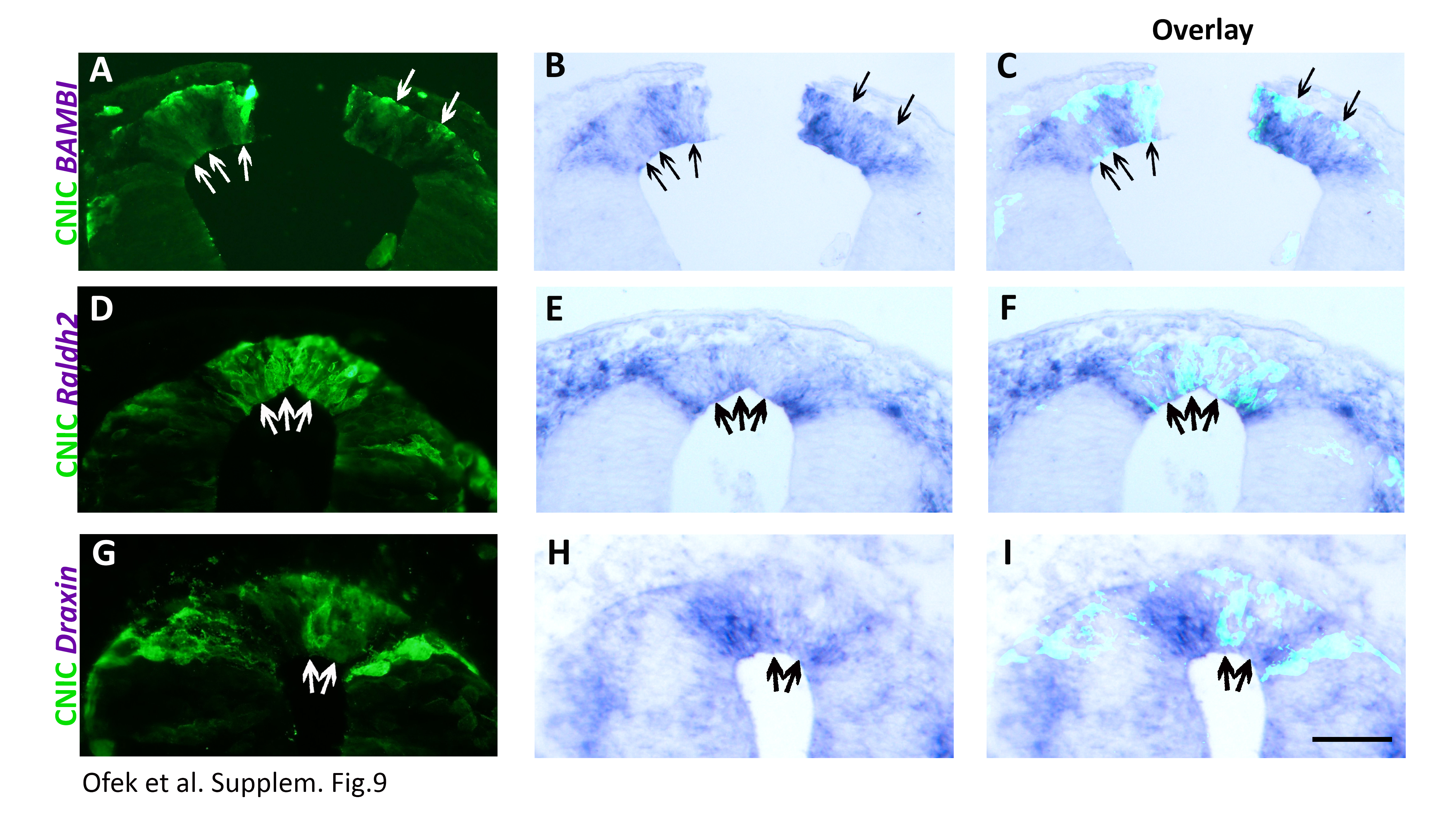

Supplement: Supplementary file 11 — Additional file 11: Fig. S9. Missexpression of active Notch1 (CNIC) in quail neural tubes prevents the upregulation of RP markers. Left column represents electroporation of CNIC-GFP (A,D,G). Middle column depicts ISH for BAMBI (N= 3), Raldh2 (N=5) or Draxin (N= 4), and right column is an overlay of the precedent, respectively. Note that CNIC-treated cells are devoid or have reduced marker expression (arrows). For control GFP see Fig. 4. Bar= 50μm. [file 12915_2021_1014_MOESM11_ESM.jpg]

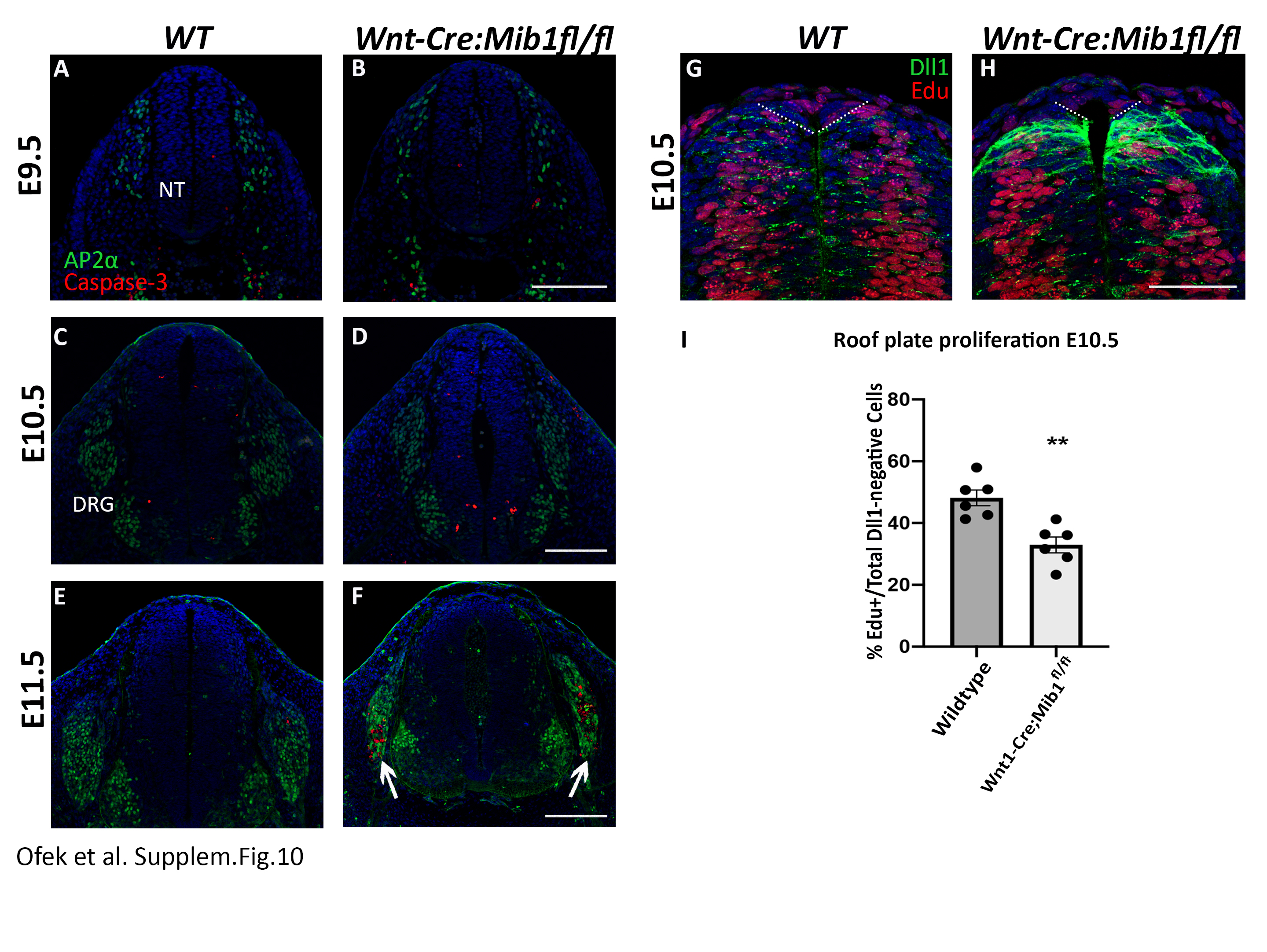

Supplement: Supplementary file 12 — Additional file 12: Fig. S10. Neural crest formation is preserved in the absence of Notch signaling. (A-F) Transverse sections through the dorsal neural tube at the level of the forelimb in wildtype (WT) and Wnt1-Cre; Mib1fl/fl mouse embryos, immunostained for AP2α labeling neural crest and dorsal root ganglion (DRG) neurons (green), and cleaved-caspase 3 labeling apoptotic cells (red). At all stages examined, no cell death is evident in the premigratory neural crest or presumptive roof plate region. At E11.5 (E, F) increased cell death is evident in the DRG of Wnt1-Cre; Mib1fl/fl embryos as previously reported (arrows). Bar = 100 μm. (G,H) Transverse sections at E10.5 immunostained for DLL1 (green) and EdU (red) following a 1hr EdU pulse to label proliferating cells. The dashed line indicates the boundary of DLL1 expression and defines the region used for quantification in (I). (I) Quantification of the number of EdU-positive proliferating cells in the Dll1-negative domain (presumptive roof plate) at E10.5, expressed as a percentage of the total number of DAPI-positive nuclei. Note the reduction in proliferating cells present in Wnt1-Cre; Mib1fl/fl embryos compared to wildtype. N=6 embryos, **p=0.0018. Bar = 50μm. [file 12915_2021_1014_MOESM12_ESM.jpg]
