# Supplementary material for: Notch signaling is a critical initiator of roof plate formation as revealed by the use of RNA profiling of the dorsal neural tube
Source: BMC Biol. 2021 Apr 23;19:84. doi: 10.1186/s12915-021-01014-3 (PMC8063321; doi:10.1186/s12915-021-01014-3)
Supplement: Supplementary file 13 — Additional file 13: Table S3. List of primers used for both quail and mouse in situ hybridization. [file 12915_2021_1014_MOESM13_ESM.docx]

**Supplementary Table S3**

| Gene name | Forward primer | Reverse primer |
| --- | --- | --- |
| qMycN | ACCACTTTTCCATCGGTCAG | ACGTGGTCCCTTAACGTGAG |
| qHes4 | TCATTCAGTCACAGCGTGGT | TTACCCCTCTCCAACACAGG |
| qRspo1 | AAACCACCGGTCTCTGTGTC | AGCAGGAGGGAAGGAAGAAG |
| qDraxin | TGTGCTGGATGTGGTTGTTT | TGGTTTGCAGAGATGCTCAC |
| qGrem1 | AGGCTGCTTTTGGAGAACAA | GAATGGGTTTTGGTTGATG |
| qCRABP1 | ACCTGGAAGATGAGGAGCAG | CACACGGTCACATACAACACC |
| qNorrin | GCACTGTCCTAAAGCAGCCT | TTCAGGCCCCGGGAGATATT |
| qLRP8 | GCCCTGAAACTGAAGACAGC | ATGCAGCTCTACTGGCGAAT |
| qZic4 | GTGAAAGGCGAAGTCTGGAG | CTTGGGTGGAAGAAGCATGT |
| qZic2 | CTCAGAGCCACCTCCTGTTC | ACTTGTCGCACATCTTGCAC |
| qMsx1 | GAGGACGAGAGCGACAAAAC | AATGGCCACAGGTTAACAGC |
| qZNF536 | TGAGAGACAGGAGCCTTGGT | TGCGACTTCTCGGTTTTTCT |
| qOLFM1 | AGCAAACTGGATCCCAACAC | ACGGGTCAGCTTTCTGCTTA |
| qINSM1 | CCAGCCACCTTCTACAGCTC | CGTTAGAGCATCTGCGATCA |
| qDll1 | TACTGCACTGAGCCGATTTG | CACCATCAGGGTTGTCAGTG |
| qBCL11B | GGACAGACCCCGACTTGTTA | ACCAAAGCCAGGATGTTCAC |
| mSlit1 | CTCTGTGTTGAGGGCTACAGTG | GGCAAATGCCATGTCGACAGTA |
| mGdf7 | CAACTCAAGGCCAGAGCTTC | TCCACCACCATGTCTTCGTA |
| mRaldh2 F | TGGAAGAATCCATCTATGAGG | AGAAACGTGGCAGTCTTGG |
| mMath1 | AGAGACCTTCCCGTCTACCC | TTCTGTGCCATCATCGCTGT |
| mLmx1a | GGGTGACGAGTTTGTCCTGA | GAGGTTCAGCACCATAGGGG |
| mRspo1 | GGATCCCAGCTAAGGTTATGGT | CAGCACTGTACTCTTCCACAGG |
| mBAMBI | GGATCGCCACTCCAGCTA | GGCCTTCAAGGGTTCCAT |
